# Prenatal Lead Levels, Plasma Amyloid β Levels, and Gene Expression in Young Adulthood

**DOI:** 10.1289/ehp.1104474

**Published:** 2012-02-07

**Authors:** Maitreyi Mazumdar, Weiming Xia, Oliver Hofmann, Matthew Gregas, Shannan Ho Sui, Winston Hide, Ting Yang, Herbert L. Needleman, David C. Bellinger

**Affiliations:** 1 Department of Neurology, Children’s Hospital Boston, Boston, Massachusetts, USA; 2 Department of Environmental Health, Harvard School of Public Health, Boston, Massachusetts, USA; 3 Department of Neurology, Brigham and Women’s Hospital, Boston, Massachusetts, USA; 4 Department of Biostatistics, Harvard School of Public Health, Boston, Massachusetts, USA; 5 Clinical Research Program, Children’s Hospital Boston, Boston, Massachusetts, USA; 6 University of Pittsburgh School of Medicine, Pittsburgh, Pennsylvania, USA

**Keywords:** Alzheimer’s disease, children, fetal basis of adult disease, human, lead

## Abstract

Background: Animal studies suggest that early-life lead exposure influences gene expression and production of proteins associated with Alzheimer’s disease (AD).

Objectives: We attempted to assess the relationship between early-life lead exposure and potential biomarkers for AD among young men and women. We also attempted to assess whether early-life lead exposure was associated with changes in expression of AD-related genes.

Methods: We used sandwich enzyme-linked immunosorbent assays (ELISA) to measure plasma concentrations of amyloid β proteins Aβ_40_ and Aβ_42_ among 55 adults who had participated as newborns and young children in a prospective cohort study of the effects of lead exposure on development. We used RNA microarray techniques to analyze gene expression.

Results: Mean plasma Aβ_42_ concentrations were lower among 13 participants with high umbilical cord blood lead concentrations (≥ 10 μg/dL) than in 42 participants with lower cord blood lead concentrations (*p* = 0.08). Among 10 participants with high prenatal lead exposure, we found evidence of an inverse relationship between umbilical cord lead concentration and expression of ADAM metallopeptidase domain 9 (*ADAM9*), reticulon 4 (*RTN4*), and low-density lipoprotein receptor-related protein associated protein 1 (*LRPAP1*) genes, whose products are believed to affect Aβ production and deposition. Gene network analysis suggested enrichment in gene sets involved in nerve growth and general cell development.

Conclusions: Data from our exploratory study suggest that prenatal lead exposure may influence Aβ-related biological pathways that have been implicated in AD onset. Gene network analysis identified further candidates to study the mechanisms of developmental lead neurotoxicity.

The weight of evidence supports an association between early-life exposure to lead and impaired cognitive function in children (Bellinger 2004; Lanphear et al. 2005; Needleman 2004). Even at low levels, childhood lead exposure results in cognitive dysfunction that persists into adulthood (Mazumdar et al. 2011). In older adults, chronic environmental exposure to lead is associated with accelerated cognitive decline (van Wijngaarden et al. 2009; Weisskopf et al. 2004, 2007; Weuve et al. 2009; Wright et al. 2003). Whether early exposure to lead has latent effects that contribute to neurodegenerative disease in old age is unknown.

Barker and Osmond (1986) demonstrated an inverse relationship between birth weight and the incidence of cardiovascular disease. The Barker hypothesis, also known as the fetal basis of adult disease (FeBAD) hypothesis, states that many adult diseases have a fetal origin (Barker 1995). According to FeBAD, injury occurring at a critical period of development could result in changes in gene expression or gene imprinting, leading to deficits that become apparent later in life.

Alzheimer’s disease (AD) is a progressive, neurodegenerative disorder that results in dementia and death. The two classical lesions of AD are *a*) neuritic plaques containing extracellular deposits of the amyloid β (Aβ) proteins and *b*) neurofibrillary tangles, which are bundles of paired, helically wound filaments inside neurons (Selkoe 2008). The leading hypothesis for AD pathogenesis suggests that accumulation of Aβ in the brain is the primary influence driving its development (Hardy and Selkoe 2002). Animal and cell culture studies have shown that lead exposure affects Aβ production (Gu et al. 2011; Huang et al. 2011).

Recent animal studies suggest the expression of AD-related genes is altered in rodents and primates exposed to lead as infants. Basha et al. (2005) exposed newborn rats to lead; at 20 months of age, the gene encoding β-amyloid precursor protein (APP) exhibited a delayed overexpression (Basha et al. 2005). The increase in APP gene expression in old age was accompanied by an elevation in Aβ in brain tissue. These changes were not seen in rats exposed to lead as adults, suggesting that early timing of lead exposure is an important determinant of gene expression and protein production. The same group also found that cynomolgus monkeys exposed to lead as infants had more neuritic plaques in their brains and exhibited higher levels of APP and Aβ than did monkeys not exposed to lead (Wu et al. 2008). In humans, case studies have reported that children who died of acute lead poisoning had neurofibrillary tangles in their brains at autopsy (Hess and Straub 1974; Niklowitz 1975; Wisniewski et al. 1979).

The long latent period between exposure and outcome poses unique challenges to the study of the FeBAD hypothesis. Most previous research on early-life environmental influences on neurodegenerative diseases has been cross-sectional or retrospective in design, thus limited in its ability to collect data on early exposures. Use of an existing cohort with banked data, ideally with biological samples, is a promising method for studying whether early-life environmental exposures result in neurodegeneration.

The Boston prospective study was one of several cross-sectional and cohort studies initiated in the late 1970s and early 1980s to study the relationship between lead and early development. Follow-up of the Boston cohort showed that the association noted between blood lead concentration and IQ at 2 years of age continued at 10 years of age (Bellinger et al. 1991). A recent report suggests that the association between early-life lead exposure and cognitive function persists into adulthood (Mazumdar et al. 2011).

The objective of this exploratory study was to assess the relationship between early-life environmental lead exposure and potential biomarkers for AD among a group of young adults who were enrolled in the Boston prospective cohort study as newborns. We also attempted to assess whether developmental exposure to lead was associated with changes in expression of genes believed to be involved in AD pathogenesis.

## Materials and Methods

*Study population.* Between August 1979 and April 1981, a cohort of 249 infants was established among babies born at the Brigham and Women’s Hospital in Boston, Massachusetts. Umbilical cord blood lead concentrations were measured, and postnatal blood lead concentrations and development were assessed at 6, 12, 18, 24, and 57 months, and again at 10 years. Follow-up of this cohort at 10 years of age included 148 children (87.6% of those considered eligible; 59.4% of the original cohort) (Bellinger et al. 1992).

In January 2009, members of the original cohort were mailed an introductory letter explaining a new study regarding early-life lead exposure and health outcomes in adulthood. Names and last known addresses were available for only the 148 participants who took part in the 10-year follow-up study. The Committee for Clinical Investigation at Children’s Hospital Boston approved the study, and each participant provided written informed consent.

*Measures of exposure, birth to 10 years.* Blood samples were obtained from umbilical cords at birth, from participants using capillary tubes at ages 6, 12, 18, and 24 months, and via venipuncture at 57 months and 10 years. Blood lead concentrations were measured in duplicate using graphic furnace atomic absorption spectrometry (Bellinger et al. 1987, 1992).

*Measurement of covariates.* At the time of enrollment in the present study, participants completed a questionnaire that gathered information on demographics, medical history, family medical history, concurrent medications, and alcohol and tobacco use. Information about other potentially important variables, including maternal medication and alcohol and tobacco use, was available in records from earlier assessments.

*Plasma A*β *subspecies isolation.* Blood used for plasma Aβ analysis was collected from the present cohort via venipuncture in EDTA tubes, processed immediately (centrifuged and aliquotted as plasma, buffy coat, and red blood cells), and then stored at –80°C. Plasma samples were sent to and analyzed in the Selkoe Laboratory at Brigham and Women’s Hospital.

Aβ_40_ and Aβ_42_ were assayed by sandwich enzyme-linked immunosorbent assays. Plates were coated with capture antibodies (2G3 for Aβ_40_ and 21F12 for Aβ_42_) in phosphate-buffered saline (PBS), incubated for 4 hr at room temperature, and then blocked with 4% Block Ace (BA; AbD Serotec, Raleigh, NC) at 4°C. Plates were washed three times with PBS containing Tween 20, and samples were freshly diluted in 0.4% BA, loaded into the wells, and incubated with antibodies for 2 hr at room temperature. Samples were then reincubated in solution containing detector biotinylated antibody (266B) for 2 hr at room temperature. Finally, samples were incubated with streptavidin alkaline phosphatase (Promega, Madison, WI) in PBS for 1 hr at room temperature and washed three times with Tris-buffered saline. After adding AttoPhos (Promega), the signal was amplified and measured with a Victor2 microtiter plate reader (PerkinElmer, Boston, MA).

*RNA preparation.* Blood used for RNA microarray analysis was collected from the present cohort via venipuncture into PAXgene tubes (QIAGEN Inc., Valencia, CA). Blood tubes were stored at –80°C until RNA extraction. Total RNA was isolated according to manufacturer protocols and purified using the PAXgene Blood RNA kit (QIAGEN Inc.). RNA extraction was performed at the General Clinical Research Center (GCRC) laboratories at Children’s Hospital Boston for the initial 12 samples. The GCRC laboratory closed during the study, and RNA extraction was performed on the remaining 37 samples at the Molecular Genetics Core Facility of the Children’s Hospital Boston Intellectual and Developmental Disabilities Research Center (IDDRC).

*Microarray hybridization.* Gene transcript expression was analyzed using Affymetrix Human Genome 1.0 ST array (Affymetrix, Santa Clara, CA). All RNA samples were analyzed at the Microarray Core Facility of the Dana-Farber Cancer Institute. Array data were retrieved and processed using the BioConductor framework, version 2.9 (Gentleman et al. 2004; http://www.bioconductor.org/), and tested for quality using the arrayQualityMetrics package (Kauffmann et al. 2009).

*Statistical analysis.* Statistical methods focused on estimating the association between early-life blood lead concentrations and plasma Aβ subspecies concentrations. We calculated correlation coefficients and fitted linear regression models with individual Aβ subspecies as the response and lead concentrations as the predictors. We fitted separate models for each measurement of lead concentration, and the average lead concentrations over the first 10 years of life were modeled as simple continuous variables and as log-transformed variables to account for heteroskedasticity and a possible nonlinear relationship between lead exposure and plasma Aβ subspecies. Outcomes were plasma Aβ_42_ concentration, Aβ_40_ concentration, and the ratio of Aβ_42_ and Aβ_40_ concentrations, which also were modeled before and after log transformation. We also stratified analyses by high and low prenatal lead levels, where high lead level was defined as > 10 μg/dL. We chose 10 μg/dL as our cutoff because this definition for high lead level was used in the original cohort study and was part of the initial sampling and recruitment strategy (Bellinger et al. 1987).

*Microarray data analysis.* RNA expression data from 49 samples were evaluated using standard quality control criteria to assess the variability across samples, for example, batch effects that may have resulted from experiments being performed on different days or by different individuals, as well as the spatial distribution of probe intensities across chips. Arrays were then background corrected and normalized with robust multichip analysis. IsoGene (Lin et al. 2007), an R statistical software package (http://www.r-project.org/), was used to identify genes with significant changes of gene expression levels in response to an increase in cord blood lead concentration at a 5% false discovery rate (FDR; determined using the Benjamini–Hochberg correction for multiple testing). A total of 245 probes mapping to 196 unique genes were considered significant using this criteria. To identify the subset of genes exhibiting monotonous changes in expression levels with increasing prenatal lead levels (at |*r*| ≥ 0.9), the 10 samples with blood prenatal blood levels > 10 μg/dL were binned into groups so that each group had approximately the same number of samples (lead exposures levels 10–12, 12–15, 15–18, 18–20 μg/dL) and analyzed in GATE (Grid Analysis of Time Series Expression) software (MacArthur et al. 2010; http://amp.pharm.mssm.edu/maayan-lab/gate.htm). Genes identified in the IsoGene analysis that passed the |*r*| ≥ 0.9 correlation filter were subsequently tested for Gene Ontology enrichment using the GeneMANIA Cytoscape plugin (Warde-Farley et al. 2010; http://www.genemania.org/plugin/). GeneMANIA extends a provided gene list with functionally similar and interacting genes to generate a functional association network. Association data include protein and genetic interactions, pathways, coexpression, colocalization, and protein domain similarity. In addition, we reviewed the literature to determine if any of the identified genes were reported to be implicated in AD biology.

## Results

We located 89 (60%) of the 148 cohort members who participated in the 10-year follow-up study. Of these, 58 enrolled in the present study. Fifty-five blood samples were available for plasma studies. The study population generally consisted of white, college-educated children with college-educated mothers. Participants were similar to members of the original cohort in terms of demographic factors, measures of socioeconomic status, blood lead history, and IQ scores in early childhood (Table 1).

**Table 1:** Characteristics of the participants at 28–30 years of age and comparison with nonparticipants.

Characteristic	Participants			Nonparticipants (n = 90)
	With plasma available (n = 55)	With RNA used in analysis (n = 36)		
Participants						
Age at testing (years)		28.9 ± 0.5		29.0 ± 0.4		—
Body mass index (kg/m2)		25.2 ± 4.5		25.0 ± 4.7		
College graduate		83.6		91.7		—
Currently smoke		18.2		19.4		—
Alcohol use > 2 drinks/week		38.1		33.3		—
Mother or father with dementia		3.4		0		—
Currently taking oral contraceptives		14.5		22.2		—
Currently taking medicine other than oral contraceptives		0		0		—
Male		47.2		36.8		53.3
White		94.5		94.7		94.4
Weeks of gestation		40.0 ± 1.9		39.8 ± 2.3		40.0 ± 1.6
Birth weight (kg)		3.4 ± 0.5		3.4 ± 0.5		3.4 ± 0.5
Blood lead concentration (μg/dL)						
Cord		6.3 ± 5.0		6.3 ± 5.4		7.9 ± 5.4
6 months		7.8 ± 5.1		7.4 ± 5.6		9.3 ± 8.0
12 months		9.8 ± 6.8		9.6 ± 7.0		9.9 ± 6.4
24 months		8.1 ± 4.3		7.7 ± 4.0		8.8 ± 5.1
4 years		6.6 ± 3.4		6.4 ± 3.9		6.1 ± 4.0
10 years		3.0 ± 2.5		2.5 ± 2.3		2.9 ± 2.4
IQ at 4 years of age		118.4 ± 14.0		117.3 ± 14.4		114.3 ± 15.4
IQ at 10 years of age		118.7 ± 14.2		116.1 ± 16.3		114.5 ± 13.8
Participants’ mothers						
Age at delivery (years)		30.6 ± 4.0		31.4 ± 4.0		30.4 ± 4.2
College graduate		60.0		72.2		62.2
Maternal IQ		124.5 ± 17.8		127.6 ± 14.1		124.9 ± 14.7
Tobacco use during pregnancy		23.6		33.3		22.5
Alcohol use during pregnancy		47.2		48.4		42.7
Data are percent yes or mean ± SD.						

*Blood lead concentration.* Mean blood lead concentration in the 55 cohort members for whom plasma samples were available was lowest at 10 years of age (3.0 μg/dL) and highest at 12 months of age (9.8 μg/dL; Table 1). Median concentrations followed a similar pattern, except median blood lead concentration was highest at 2 years of age [8.1 μg/dL; see Supplemental Material, Figure 1 (http://dx.doi.org/10.1289/ehp.1104474)]. The highest lead concentrations were seen in infancy and early childhood, possibly reflecting greater lead intake through hand-to-mouth activity and higher exposures in that era.

**Figure 1:**
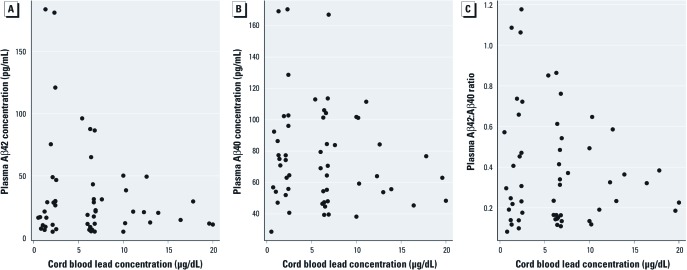
Scatterplots of prenatal (umbilical cord) lead concentrations versus plasma Aβ subspecies concentrations (*n* = 55 plasma samples). Aβ_42_ (*A*) and Aβ_40_ (*B*) at 28 years of age and Aβ_42_:Aβ_40_ ratio at 29 years of age (*C*).

Aβ *analysis.* Scatterplots of prenatal lead concentration, Aβ_42_ concentration, and Aβ_42_:Aβ_40_ ratio suggest an inverse relationship between prenatal blood lead concentration and plasma Aβ subspecies in adulthood (Figure 1). The mean ± SD Aβ_42_ concentration among participants with prenatal lead exposure < 10 μg/dL was 36.6 ± 43.1 pM, compared with 22.8 ± 14.7 pM among participants with higher prenatal lead exposure (*p* = 0.08; Figure 1). The Aβ_42_:Aβ_40_ ratio showed a similar pattern between the two groups, with a ratio of 0.40 ± 0.30 for participants with prenatal lead concentration ≥ 10 μg/dL and 0.32 ± 0.17 for participants with prenatal blood lead levels < 10 μg/dL (*p* = 0.27). Models of untransformed and log-transformed lead concentrations at different time points did not indicate clear or consistent associations with plasma Aβ_42_ or the Aβ_42_:Aβ_40_ ratio. Visual inspection of the scatterplots among participants with high prenatal lead exposure reveals an apparent inverse relationship between umbilical cord lead concentration and plasma Aβ_42_ concentration as well as plasma Aβ_42_:Aβ_40_ ratio (Figure 1).

*AD-related gene expression.* Forty-nine samples were available for RNA analysis, and 36 samples that met standard quality control criteria were included in analyses. All of the 36 samples used in the analyses were processed at the IDDRC. The expression of 196 genes was significantly associated (5% FDR) with increasing cord blood lead level. Because of the small sample size, an additional filter was applied, retaining only genes with a correlation of |*r*| ≥ 0.9 with an increasing (or decreasing) trend between cord blood levels of 10–20 μg/dL. The 39 retained genes [listed in Supplemental Material, Table 1 (http://dx.doi.org/10.1289/ehp.1104474)] exhibited a much higher level of variation in gene expression in relation to cord blood level concentrations < 10 μg/dL (*n* = 28) compared with concentrations > 10 μg/dL (*n* = 10; data not shown).

**Figure 2:**
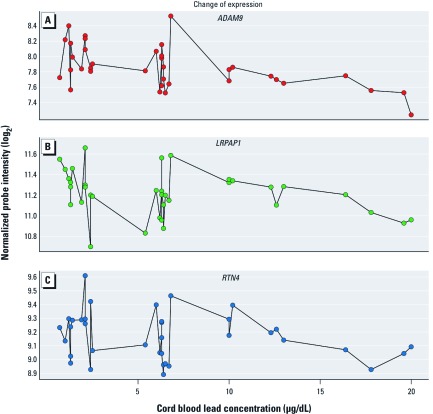
Gene expression levels for selected AD-related genes: normalized probe intensities for *ADAM9* (*A*), *RTN4* (*B*), and *LRPAP1* (*C*) at 29 years of age in relation to prenatal (umbilical cord) blood lead concentrations. Data points from samples with cord blood lead level concentrations < 10 μg/dL (*n* = 26) were not used to identify genes whose expression was significantly associated with blood lead levels > 10 μg/dL (*n* = 10) and are plotted for reference only.

Three of the 39 identified genes encode proteins that have been reported to affect Aβ production and deposition in the brain; ADAM metallopeptidase domain 9 (*ADAM9*), low-density lipoprotein receptor-related protein associated protein 1 (*LRPAP1*) and reticulon 4 (*RTN4*). Figure 2 shows normalized probe intensities (measures of relative gene expression) of these genes in relation to prenatal blood lead concentrations for individual participants.

Genes whose expression showed an increasing or decreasing trend in relation to prenatal lead level were found to interact directly or indirectly in physical interaction and coexpression networks [see Supplemental Material, Figure 2 (http://dx.doi.org/10.1289/ehp.1104474)]. Although the network analysis did not exhibit a direct involvement with amyloid pathways, it identified enrichment of genes associated with the (negative) regulation of neurogenesis, nerve growth, and general cell development (Table 2).

**Table 2:** Network analysis representing functional association of 39 genes with altered expression at prenatal lead levels > 10 μg/dL.

Gene Ontology biological process	Q-value	Count [annotated (overall)]
Neuron development				
Negative regulation of axonogenesis		0.00080		4 (13)
Negative regulation of cell projection organization		0.00328		4 (21)
Negative regulation of neurogenesis		0.00634		4 (27)
Nerve growth factor receptor signaling pathway		0.00990		7 (215)
Regulation of axonogenesis		0.00990		4 (34)
Regulation of neuron projection development		0.08320		4 (63)
Cell development				
Negative regulation of cell development		0.01040		4 (36)
Negative regulation of cell differentiation		0.10500		5 (148)
Immune response				
Response to abiotic stimulus		0.08320		6 (218)
T-cell receptor signaling pathway		0.08490		4 (67)
Data are Gene Ontology categories (http://www.geneontology.org/) and Q-values (minimum FDR at which the test is considered significant) from an FDR-corrected hypergeometric test for enrichment. Q-Values were estimated using the Benjamini–Hochberg procedure. Counts reflect genes with this annotation in the query set (39 genes of interest identified by differential monotonous change of expression in response to lead exposure) compared with the overall number of genes in the GeneMANIA background set with this annotation and at least one interaction (numbers in parentheses).				

## Discussion

Two observations from this study support the hypothesis that early-life (prenatal) lead exposure influences the biological pathways believed to be involved in AD. First, participants with higher prenatal lead exposure (umbilical cord lead concentration > 10 μg/dL) had lower mean plasma Aβ_42_ concentrations than did participants with low and moderate prenatal lead levels. Data from participants with higher prenatal lead levels showed an apparent inverse relationship between umbilical cord lead concentration and both plasma Aβ_42_ concentration and plasma Aβ_42_:Aβ_40_ ratios.

The second finding is that among the 10 participants with higher prenatal lead exposure (> 10 μg/dL), there were inverse relationships between umbilical cord lead concentrations and expression of *ADAM9*, *RTN4*, and *LRPAP1*, genes whose products are believed to affect Aβ production and deposition in the brain (Allinson et al. 2003; Cong and Jia 2011; He et al. 2004; Hyman et al. 2000; Murayama et al. 2006). The expression of 39 genes appeared to be associated with prenatal blood lead levels within this subgroup, but these three genes have been previously found relevant to AD pathogenesis.

The product of *ADAM9* is a member of the ADAM (a disintegrin and metalloprotease domain) family. Members of this family are membrane-anchored proteins that have been implicated in a variety of biological processes, including neurogenesis. The product of *ADAM9* is believed to act as an α-secretase and cause nonamyloidogenic cleavage of APP (Allinson et al. 2003; Cong and Jia 2011; Deuss et al. 2008).

The product of *RTN4* is a potent neurite outgrowth inhibitor that may also help block the regeneration of the central nervous system in higher vertebrates. RTN4 is also implicated in the amyloid cascade. Animal studies have suggested that the product of *RTN4* decreases cleavage of APP to its amyloidogenic products (He et al. 2004; Murayama et al. 2006).

Low-density lipoprotein receptor-related protein (LRP) is thought to play an important role in determining the balance between Aβ synthesis and clearance mechanisms (Hyman et al. 2000). The product of the *LRPAP1* gene blocks ligand-binding sites of LRP and interferes with Aβ clearance. In accordance with its role in the amyloidogenic pathway, variation in *LRPAP1* has been associated with the risk of developing AD (Sanchez et al. 2001).

Although gene network analysis did not suggest a direct involvement with amyloid pathways, several genes that exhibited decreased expression in relation to prenatal lead concentration are involved in the (negative) regulation of neurogenesis, nerve growth, and general cell development. This provides additional support to the hypothesis that developmental exposure to lead can cause changes in expression for genes important in neurological development and disease. Our findings were similar to those seen in zebrafish embryos (Peterson et al. 2011) after researchers exposed them to a sublethal dose of lead and analyzed global transcriptional alterations.

The known association between AD and both limited education and occupational attainment (Cobb et al. 1995; Snowdon et al. 2000; Stern et al. 1994), often referred to as the cognitive reserve hypothesis, suggests that risk factors for AD may be established early in life. The cognitive reserve hypothesis posits that higher levels of education, or other measures of cognitive ability early in life, provide protection against cognitive decline, suggesting that early education leads to the development of efficient and/or flexible neural networks more capable of coping with the disruption imposed by brain pathology (Stern 2009). An alternative explanation is that the same environmental influences that result in poor cognition in childhood and early adulthood have persistent and/or latent effects that contribute to the development of dementia during old age.

Our study used prospectively collected lead exposure information from a cohort study started > 30 years ago. The long latent period between exposure and outcome poses significant challenges to the study of the FeBAD hypothesis, and the imprecision of exposure classification based on ecological data or recall is one of the biggest criticisms of this work (Joseph and Kramer 1996). Our study minimized the uncertainty around exposure assessment by using samples that were collected and analyzed in real time.

Our study has a number of limitations, the most important of which is a small number of participants. Specifically, we had only 13 participants with prenatal blood lead levels > 10 μg/dL (including 10 with gene expression data), which turned out to be the exposure level of greatest interest. The Boston cohort was specifically assembled to investigate the cognitive effects of what was then considered to be low-level lead exposure. Repeating this analysis among participants from cohorts with higher prenatal lead levels may provide more data around the exposure levels of interest, therefore providing more power and more precise estimates of association.

Another important limitation is the use of plasma measurements of Aβ instead of cerebrospinal fluid measurements or neuropathological changes (as would be seen at autopsy) to define the outcome. The published data on plasma Aβ levels in AD are contradictory, with plasma Aβ levels reported to be higher (Kosaka et al. 1997; Sobow et al. 2005), lower (Buerger et al. 2009; Cosentino et al. 2010), or unchanged (Abdullah et al. 2007; Fukumoto et al. 2003; Giedraitis et al. 2007; Tamaoka et al. 1996) in cases with AD compared with controls. A recent meta-analysis found that in cross-sectional studies, AD patients in their 60s and 70s had lower Aβ_42_ plasma concentrations compared with cognitively normal individuals (Song et al. 2011), although this pattern was not seen in longitudinal studies. A proposed mechanism for the discrepancy between high Aβ levels seen in the brains of AD cases and low Aβ subspecies levels in plasma is that there is compartmentalization of Aβ peptides in the brain among patients with AD (Schupf et al. 2008). There are no reports regarding the association between plasma Aβ among young adults and the development of AD.

An additional limitation of our study is that we did not measure blood lead levels at the same time that we measured plasma Aβ and gene expression levels. In a recent pooled analysis, current blood lead levels and average lifetime estimates were stronger predictors of intellectual deficits in late childhood than was early childhood lead concentration (Lanphear et al. 2005). In older adults, however, recent studies found that current blood lead levels were not associated with cognitive function in adults, suggesting that early-life or accumulated exposures may be etiologically more important (van Wijngaarden et al. 2011). Future studies would be strengthened if associations are estimated between outcome and current blood lead levels, as well as early blood lead levels.

An additional concern is the use of multiple testing in microarray experiments. Given thousands of genes, it is possible that the ones we found to be associated with prenatal lead exposure were so associated by chance (Ioannidis 2005). We corrected for this limitation by looking at genes with continuous changes at higher prenatal levels (following the trend seen in scatterplots) and also by comparing these with genes implicated in AD pathogenesis. However, our findings should be considered preliminary.

If early-life lead exposure influences biological processes involved in AD pathogenesis, the implications for public health practice are substantial. Lead exposure in the United States reached its peak in the 1970s, and the children exposed at that time, now adults, may be at increased risk for neurodegenerative disease. If similar results are achieved in a larger cohort, our study could provide support for screening strategies that identify adults who may be at higher risk for neurodegenerative disease because of childhood exposures, as well as identify an opportunity for disease prevention.

## Conclusions

Data from our study suggest that early-life lead exposure may influence the biological pathways that have been implicated in AD in old age. Gene network analysis identified further candidates for the study of the mechanisms of developmental lead neurotoxicity in humans.

## Supplemental Material

(336 KB) PDF
