# Supplementary material for: Prenatal Lead Levels, Plasma Amyloid β Levels, and Gene Expression in Young Adulthood
Source: Environ Health Perspect. 2012 Feb 7;120(5):702–7. doi: 10.1289/ehp.1104474 (PMC3346789; doi:10.1289/ehp.1104474)
Supplement: (336 KB) PDF [file ehp.1104474.s001.pdf]

SUPPLEMENTAL MATERIAL

to accompany

Prenatal Lead Levels, Plasma Amyloid β levels and Gene Expression in Young Adulthood

Maitreyi Mazumdar, Weiming Xia, Oliver Hofmann, Matthew Gregas, Shannan Ho Sui, Winston Hide, Ting Yang, Herbert L. Needleman and David C. Bellinger

Table of Contents

Figure 1. Blood lead concentrations at each measuring point 2
Figure 2. Gene network figure 4
Table 1. Genes altered at prenatal lead concentration > 10 μg/dL 6

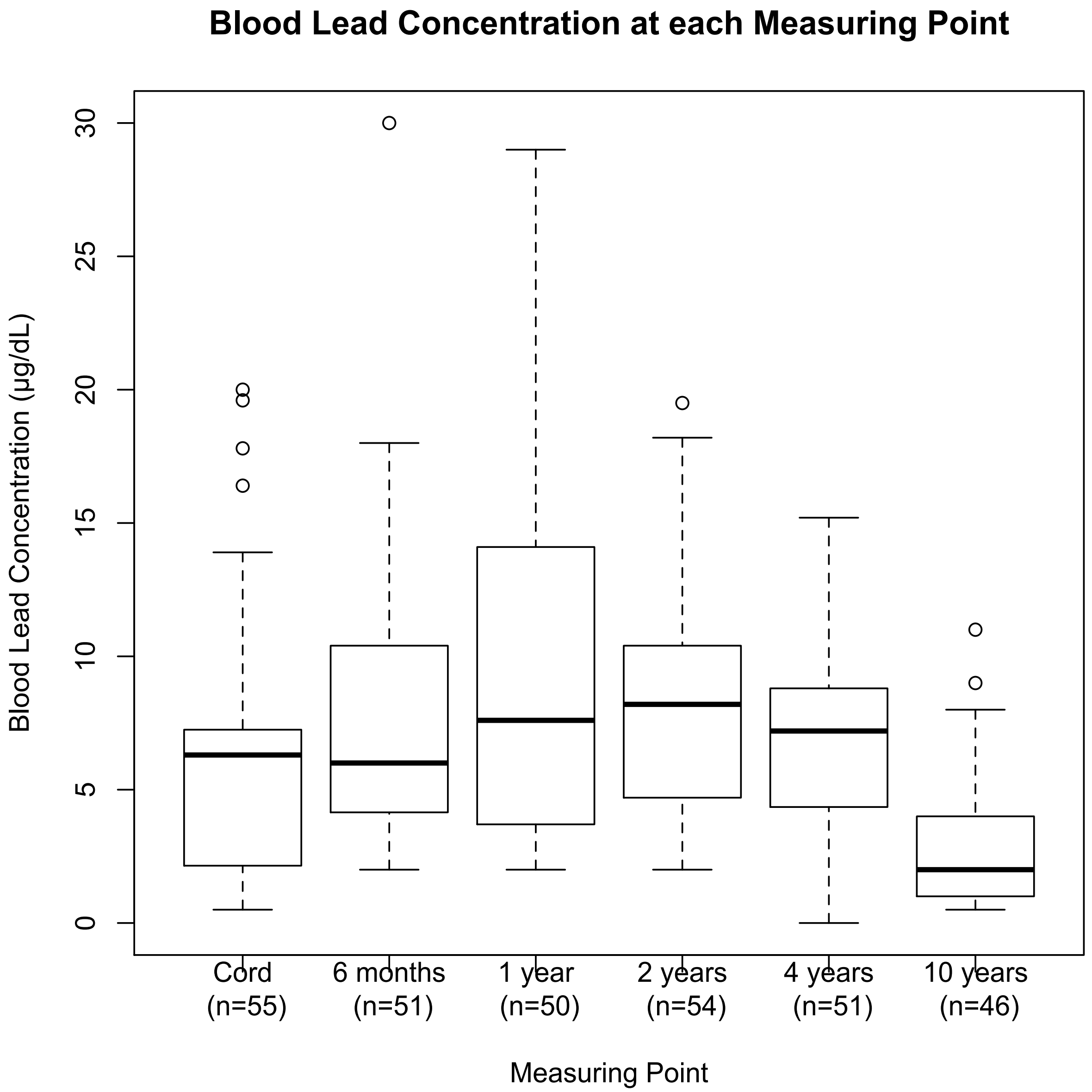
Blood Lead Concentration at each Measuring Point
Blood Lead Concentration (µg/dL)
0
5
10
15
20
25
30
Cord (n=55)
6 months (n=51)
1 year (n=50)
2 years (n=54)
4 years (n=51)
10 years (n=46)
Measuring Point

Supplemental Material, Figure 1. Blood lead concentration at each measuring point. In each box plot, the median value is indicated by the center horizontal line and the 25th and 75th percentiles are indicated by the lower and upper horizontal lines, respectively. The vertical lines represent 1.5 times the interquartile range, and circles represent outliers (i.e. values that are more than 1.5 times the interquartile range). To convert values for lead to micromoles per liter, multiply by 0.0483.

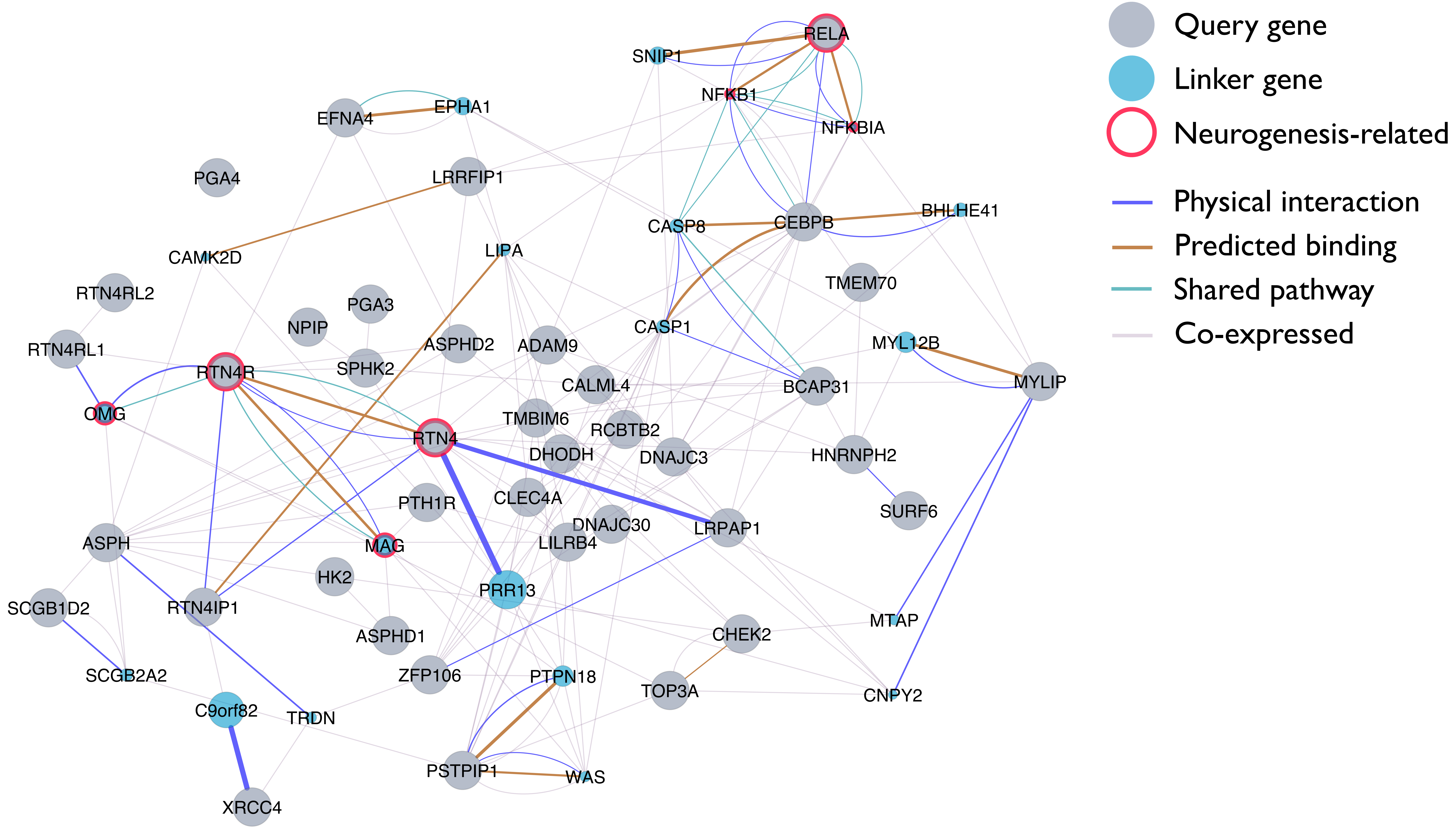

Query gene
Linker gene
Neurogenesis-related
Physical interaction
Predicted binding
Shared pathway
Co-expressed
RELA
SNIP1
NFKB1
NFKBIA
EFNA4
EPHA1
PGA4
LRRFIP1
CAMK2D
LIPA
CASP8
CEBPB
BHLHE41
TMEM70
RTN4RL2
PGA3
NPIP
ASPHD2
ADAM9
CASP1
MYL12B
RTN4RL1
RTN4R
SPHK2
CALML4
BCAP31
MYLIP
OMG
TMBIM6
RCBTB2
RTN4
DHODH
DNAJC3
HNRNPH2
PTH1R
CLEC4A
SURF6
DNAJC30
LRPAP1
ASPH
MAG
LILRB4
HK2
PRR13
SCGB1D2
RTN4IP1
ASPHD1
CHEK2
MTAP
SCGB2A2
ZFP106
PTPN18
TOP3A
CNPY2
C9orf82
TRDN
PSTPIP1
WAS
XRCC4

Supplemental Material, Figure 2. Gene network figure based on 10 subjects with prenatal lead concentration ≥ 10 µg/dL. Network representation based on GeneMania (Warde-Farley et al, default parameters) representing functional association of 39 genes ('query genes') identified by IsoGene analysis as having decreased expression with prenatal lead levels > 10 µg/dL and ten additional related genes based on functional association ('linker genes'). Association data generated from 144 public data sets include protein and genetic interactions, pathways, co-expression, co-localization and protein domain similarity. Node side reflects GeneMania scores (larger nodes are more relevant).

Warde-Farley D, Donaldson SL, Comes O, Zuberi K, Badrawi R, Chao P, Franz M, Grouios C, Kazi F, Lopes CT, Maitland A, Mostafavi S, Montojo J, Shao Q, Wright G, Bader GD, Morris Q

Supplemental Material, Table 1. Genes altered at prenatal lead concentration < 10 μg/dL

Symbol	Gene name
ADAM9	ADAM metallopeptidatse domain 9
ASPH	aspartate beta-hydroxylase
ASPHD1	aspartate beta-hydroxylase domain containing 1
ASPHD2	aspartate beta-hydroxylase domain containing 2
BCAP31	B-cell receptor-associated protein 31
CALML4	calmodulin-like 4
CEBPB	CCAAT/enhancer binding protein, beta
CHEK2	CHK2 checkpoint homolog
CLEC4A	C-type lectin domain family 4, member A
DHODH	dihydroorotate dehydrogenase
DNAJC3	DNAJ (HSP40) homolog, subfamily C, member 3
DNAJC30	DNAJ (HSP40) homolog, subfamily C, member 30
EFNA4	ephrin-A4
HK2	hexokinase 2
HNRNPH2	heterogeneous nuclear ribonucleoprotein H2
LILRB4	leukocyte immunoglobuin-like receptor, subfamily B, member 4
LRPAP1	lipoprotein receptor-related protein associated protein 1
LRRFIP1	leucine rich repeat (in FLII) interacting protein 1
MYLIP	myosin regulatory light chain interacting protein
NPIP	nuclear pore complex interacting protein
PGA3	pepsinogen 3, group I (pepsinogen A)
PGA4	pepsinogen 4, group I (pepsinogen A)
PSTPIP1	proline-serine-threonine phosphatase-interacting protein 1
PTH1R	parathyroid hormone 1 receptor
RCBTB2	regulator of chromosome condensation and BTB domain containing protein 2
RELA	v-rel reticuloendotheliosis viral oncogene homolog a (avian)
RTN4	reticulon 4
RTN4IP1	reticulon 4 interacting protein 1
RTN4R	reticulon 4 receptor
RTN4RL1	reticulon 4 receptor-like 1
RTN4RL2	reticulon 4 receptor-like 2
SCGB1D2	secretoglobin, family 1D, member 2
SPHK2	spinhgosine kinase 2
SURF6	surfeit 6
TMBIM6	transmembrane BAX inhibitor motif containing 6
TMEM70	transmembrane protein 70
TOP3A	topoisomerase (DNA) III alpha
XRCC4	X-ray repair complementing defective repair in Chinese hamster cells 4
ZFP106	zinc finger protein 106 homolog
